# Comments on “Association of excessive mobile phone use during pregnancy with birth weight: an adjunct study in Kumamoto of Japan Environment and Children’s Study”

**DOI:** 10.1186/s12199-017-0674-z

**Published:** 2017-09-16

**Authors:** Ghazal Mortazavi, S.A.R. Mortazavi, S.M.J. Mortazavi

**Affiliations:** 1 Ionizing and Non-ionizing Radiation Protection Research Center (INIRPRC), Shiraz University of Medical Sciences, Shiraz, Iran; 2 Student Research Committee, School of Medicine, Shiraz University of Medical Sciences, Shiraz, Iran; 3 Diagnostic Imaging Center, Fox Chase Cancer Center, Philadelphia, PA 19111 USA

**Keywords:** Mobile phone use, Electromagnetic fields, Pregnancy, Birth weight

## Abstract

We have read with interest the article by Lu et al. entitled “Association of excessive mobile phone use during pregnancy with birth weight: an adjunct study in Kumamoto of Japan Environment and Children’s Study” published recently in the Environmental Health and Preventive Medicine. Although this paper addresses a very challenging issue, it has some shortcomings. Mortazavi et al. have previously studied the effects of ionizing and non-ionizing radiation on birth weight of newborns and found no statistical significant differences between the mean weight of newborns whose mothers had been exposed to electromagnetic fields (EMF) generated by mobile phones and those of non-exposed mothers. The study performed by Lu et al. cannot answer this very key question that whether ordinary use of mobile phone during pregnancy can lead to low birth weight. The origin of the controversy between the findings of these two studies and the shortcomings of the article by Lu et al. are discussed.

This letter is regarding the Environmental Health and Preventive Medicine article by Lu et al. entitled “Association of excessive mobile phone use during pregnancy with birth weight: an adjunct study in Kumamoto of Japan Environment and Children’s Study” [1]. This paper claims that excessive mobile phone use during pregnancy may be a risk factor for lower birth weight “*The mean infant birth weight was lower in the excessive use group than in the ordinary use group, and the frequency of infant emergency transport was significantly higher in the excessive use group than in the ordinary use group*”. The study performed by Lu et al. has some major shortcomings. The effects of ionizing and non-ionizing radiation on birth weight of newborns have been previously studied by Mortazavi et al. These researchers in 2013 showed that there were no statistical significant differences between the mean weight of newborns whose mothers had been exposed to electromagnetic fields (EMF) generated by mobile phones, cordless phones, and cathode ray tubes (CRT) and those of non-exposed mothers [2]. In the study performed by Mortazavi et al., 52.75% had made use of cell phones during their pregnancy. The mean birth weight of newborns to mothers making use of cell phones was 3126.84 ± 509.39 g and that of newborns to mothers not using cell phones was 3098.44 ± 51.22 g. This difference was not statistically significant. Moreover, 78.5% had never made use of home cordless phones during their pregnancy. The mean birth weight of newborns to these mothers was 3113.31 ± 511.47 g and the mean birth weight of newborns to mothers who had used such phones during their pregnancy was 3101.32 ± 505.07 g. This difference was not statistically significant.

Finally, 84.5% had never used CRT monitors during their pregnancy. The mean birth weight of newborns to such mothers was 3108.32 ± 516.89 g and newborns to mothers using such devices was 3126.69 ± 466.69 g. Again, no statistically significant difference was found between these two groups.

In this light, our findings are not in line with those reported by Lu et al. Searching for the source of this discrepancy reveals that in the study performed by Lu et al., the mean birth weight of 415 ordinary users (90.02% of the 461 study participants) is compared with that of 46 excessive users (9.98% of the participants). In this light, this very challenging key question that whether ordinary use of mobile phone during pregnancy (the actual scenario for more than 90% of the mothers participated in this study) can lead to low birth weight, remains unanswered.

The 2nd shortcoming of this paper comes from this point that the 46 (9.98%) excessive users in the study performed by Lu et al. were possibly among the people who more experience stress and anxiety due to problems such as turbulent marriage and stressful jobs. It is worth mentioning that positive associations between “high” compared to “low mobile phone use” and current stress, sleep disturbances, and symptoms of depression have been reported by Thomee et al. [3].

## Abbreviations

CRT: Cathode ray tube
EMF: Electromagnetic fields
