# Supplementary material for: Comments on “Association of excessive mobile phone use during pregnancy with birth weight: an adjunct study in Kumamoto of Japan Environment and Children’s Study”
Source: Environ Health Prev Med. 2017 Sep 16;22:67. doi: 10.1186/s12199-017-0674-z (PMC5664569; doi:10.1186/s12199-017-0674-z)
Supplement: Related texts

# Reply: Authors’ response

**Authors:** Xi Lu, Masako Oda, Takashi Ohba, Hiroshi Mitsubuchi, Shota Masuda, Takahiko Katoh

We thank the research group for their interest in our study titled “Association of excessive mobile phone use during pregnancy with birth weight: an adjunct study in Kumamoto of Japan Environment and Children’s Study” as well as their highly relevant comments on our work. Please find below our responses on the points raised by the commenters:

1. Based on our title [1], our study aimed to determine the association between excessive mobile phone use and neonatal birth weight and infant health status. The purpose of this study does not include the study of the effects of the phone’s electromagnetic fields on these two outcomes. Excessive mobile phone use is recognized as a consequential social problem; however, its effects on the fetus during pregnancy remain to be fully determined. Thus, we just focused on the differences in the neonatal birth weight and infant health status between mothers who were excessive mobile users and those who were normal mobile users.

Although the goal of our study is not to determine the effects of the electromagnetic fields on the two previously mentioned outcomes, we also checked the daily designated spot, power state during sleep, and location of the mobile phone when sleeping during pregnancy to prevent bias (Table 1). In terms of the designated spot for mobile phone, the excessive mobile use group more frequently placed the mobile phone in the trouser or shirt pocket than the ordinary mobile use group (*P* < 0.05). However, no statistically significant differences in the power state and location of the mobile phone were observed between the two groups. Additionally, as pointed out in our discussion, “We also found that the excessive use group preferred to place the mobile phone in the trouser or shirt pocket, indicating that the excessive use group preferred to have the mobile phone in an easily accessible location.” Therefore, our finding suggests that excessive mobile phone use during pregnancy may be a risk factor for low neonatal birth weight. This finding is not against with that of Mortazavi et al. [2]. However, one key issue must be raised in the study of Mortazavi et al., that is, the exclusion of mothers with addiction from the data analysis (no further detail on the definition of addiction was provided) [2]. This exclusion may be the reason for the absence of any statistically significant difference in the newborns’ birth weights between mothers who were exposed to radiation and those who were not.

2. As previously mentioned, the objective of our study was to determine the differences in the neonatal birth weight and infant health status between mothers who were excessive mobile phone users and those who were normal mobile phone users. Hence, our study sample included mothers who were “excessive mobile phone users.” Some studies already reported that excessive mobile phone use was correlated with sleeping problems, stress, depression, and other mental health issues [4–6]. These findings were also reflected in our discussion: “Our study suggests that excessive mobile phone use during pregnancy may cause mental problems, such as anxiety and depression, and health problems, such as sleep problems and sleeplessness.”
